# Neighborhood segregation and cancer prevention guideline adherence in US Hispanic/Latino adults: Results from the HCHS/SOL

**DOI:** 10.3389/fonc.2022.1024572

**Published:** 2022-12-19

**Authors:** Margaret S. Pichardo, Catherine M. Pichardo, Gregory A. Talavera, Linda C. Gallo, Sheila F. Castañeda, Daniela Sotres-Alvarez, Yamile Molina, Kelly R. Evenson, Martha L. Daviglus, Lifang Hou, Brian Joyce, Larissa Aviles-Santa, Jesse Plascak

**Affiliations:** ^1^ Department of Psychology, San Diego State University, San Diego, CA, United States; ^2^ Department of Chronic Disease Epidemiology, Yale School of Public Health, Yale University, New Haven, CT, United States; ^3^ Department of Surgery, Hospital of the University of Pennsylvania, Penn Medicine, Philadelphia, PA, United States; ^4^ Department of Psychology, University of Illinois at Chicago, Chicago, IL, United States; ^5^ Division of Cancer Control and Population Sciences, National Cancer Institute, Bethesda, MD, United States; ^6^ Department of Biostatistics, University of North Carolina at Chapel Hill, Chapel Hill, NC, United States; ^7^ Division of Community Health Sciences, School of Public Health, University of Illinois at Chicago, Chicago, IL, United States; ^8^ Department of Epidemiology, Northwestern University, Chicago, IL, United States; ^9^ Institute for Minority Health Research, University of Illinois at Chicago, Chicago, IL, United States; ^10^ Department of Preventive Medicine, Northwestern University, Chicago, IL, United States; ^11^ National Institute on Minority Health and Health Disparities, Bethesda, MD, United States; ^12^ Division of Cancer Prevention and Control, Ohio State University, Columbus, OH, United States

**Keywords:** neighborhood segregation, obesity, diet, alcohol intake, physical activity, cancer prevention guidelines, Hispanic/Latino

## Abstract

**Background:**

Adherence to the American Cancer Society (ACS) guidelines for cancer prevention is associated with a lower risk of cancer and mortality. The role of neighborhood segregation on adherence to the guidelines among Hispanic/Latino adults is relatively unexplored.

**Materials and methods:**

The Hispanic Community Health Study/Study of Latinos is a community-based prospective cohort of 16,462 Hispanic/Latino adults, ages 18-74 years enrolled in 2008-2011 from the Bronx, Chicago, Miami and San Diego. Dimensions of neighborhood segregation were measured using 2010 United States’ census tracts:—evenness (the physical separation of a group), exposure (the propensity for contact between groups), and their joint effect (hypersegregation). ACS guideline adherence levels – low, moderate, high – were created from accelerometry-measured physical activity, dietary intake, alcohol intake, and body mass index. Weighted multinominal logistic regressions estimated relative risk ratios (RRR) and 95% confidence intervals (CI) for guideline adherence levels and its components.

**Results:**

Hispanic/Latino adults were classified as low (13.7%), moderate (58.8%) or highly (27.5%) adherent to ACS guidelines. We found no evidence of an association between segregation and overall guideline adherence. Exposure segregation associated with lower likelihood of moderate adherence to alcohol recommendations (RRR_moderate vs. low_:0.86, 95%CI:0.75-0.98) but higher likelihood for diet recommendations (RRR_moderate vs. low_:1.07, 95%CI:1.01-1.14). Evenness segregation associated with lower likelihood of high adherence to the physical activity recommendations (RRR_high vs. low_:0.73, 95%CI:0.57-0.94). Hypersegregation was associated with individual guideline components.

**Conclusion:**

We found evidence of a cross-sectional relationship between neighborhood segregation and ACS cancer prevention guideline components, but not with overall ACS guideline adherence.

## Introduction

Prevalence of obesity, a disease identified in the etiology of at least 13 cancers and cancer sites (known as obesity-related cancers), remains high among U.S. Hispanic/Latino adults (1, 2). Adherence to the American Cancer Society (ACS) Guidelines on Nutrition and Physical Activity for Cancer Prevention (3, 4), which include maintaining a healthy weight throughout life, engaging in at least 150-300 minutes of moderate to vigorous physical activity every week, increasing intake of fruits, vegetables, whole grains, and reducing intake of red and processed meats, refined grains and alcohol, may reduce the risk of many obesity-related cancers (5, 6). Yet, adherence levels remain low among Hispanic/Latino adults (7, 8). For Hispanic/Latinos, the manifestation of structural racism—the intersection of low socioeconomic status and high race- and economic-based residential segregation—may contribute to poor energy balance and increase risk of developing obesity-related cancers (9–12), perpetuating cancer inequities (13).

The construct of structural racism is often operationalized as neighborhood racial-ethnic segregation and poverty; and evidence suggest that racial and ethnic segregation is particularly exacerbated by neighborhood poverty (14, 15). Segregation is formally measured using five dimensions as developed by Massey and Denton (16): evenness (the spatial distribution of a group), exposure (the propensity for contact between groups), clustering (groups of interest located in close proximity or neighboring areas), centralization (the extent to which a group resides in or near the center of an urban area), concentration (the relative amount of physical space a group occupies). High levels across more than one dimension is known as hypersegregation.

The literature on segregation using these formal, well-established measures of segregation among Hispanic/Latinos is limited. Systematic reviews of segregation and obesity (17) and segregation and cancer (18) note an overreliance of the literature on informal and non-valid, measures of segregation such as racial/ethnic density/composition, which does not reflect the distribution of racial and ethnic groups across space nor compares racial/ethnic composition between the neighborhood of interest to surrounding areas. The literature that incorporates formal segregation measures has predominantly focused on only the exposure dimension—measured by the isolation index—and its link to obesity (19–25). While majority of studies do not distinguish ‘segregation’ from ‘ethnic enclave’ methologically; conceptually the literature attempts to identify ‘ethnic enclave’ as a health promoting factor linked to social capital. Herein, we operationalize and conceptualize segregation in the context of health disparities according to White and Borrell; and we consider only segregation measures developed by Massey and Denton as ‘formal’ and other measures as ‘informal’ (26).

Regardless of the segregation measure used, studies have shown that neighborhoods with high Hispanic/Latino segregation (i.e., commonly referred to as ethnic enclave) have more obesogenic features (e.g., reduced opportunities and infrastructures for physical activity, lack of safety, low walkability, and fewer recreational resources) (27–31) and lower access to markets or stores with affordable healthy foods (30, 32), and fresh fruits and vegetables (33). These features in turn may increase risk of obesity (12, 34) among the population.

Beyond obesity, few segregation studies have examined other lifestyle behaviors related to cancer [e.g, diet quality, physical activity, and alcohol intake (33, 35)]. Moreover, none have examined segregation in relation to overall lifestyle patterns. Hispanic/Latino adults residing in segregated areas (i.e., ethnic enclaves) may have fewer opportunities to engage in the full range of healthful behaviors to prevent cancer, in concordance with ACS guidelines.

To better understand the potential mechanisms that contribute to poor energy balance and ultimately, cancer health inequities seen for Hispanic/Latino communities, we examined cross-sectional associations between neighborhood segregation and adherence to the ACS lifestyle guidelines in the Hispanic Community Health Study/Study of Latinos (HCHS/SOL).

## Materials and methods

### Study population

The HCHS/SOL is a longitudinal community-based cohort study that recruited between 2008 and 2011 (36). A total of 16,415 non-institutionalized Hispanic/Latino adults (aged 18–74 years) were enrolled in Miami, FL; San Diego, CA; Chicago, IL; and the Bronx, NY from areas with high concentrations of Hispanic/Latino residents and low residential mobility to maximize retention rates (36). Participants self-identified heritage as Cuban (n = 2,348), Puerto Rican (n = 2,728), Dominican (n = 1,473), Mexican (n = 6,472), Central American, (n = 1,732), and South American (n = 1,702). At baseline, participants completed questionnaires with trained bilingual interviewers to assess lifestyle, anthropometric, and sociodemographic characteristics. Baseline home addresses were geocoded at the census tract and linked to 2010 U.S. Census tract neighborhood indicators from the IPUMS National Historical Geographic Information System (NHGIS) (37).

### Neighborhood segregation: Formal measures

**Figure 1:**
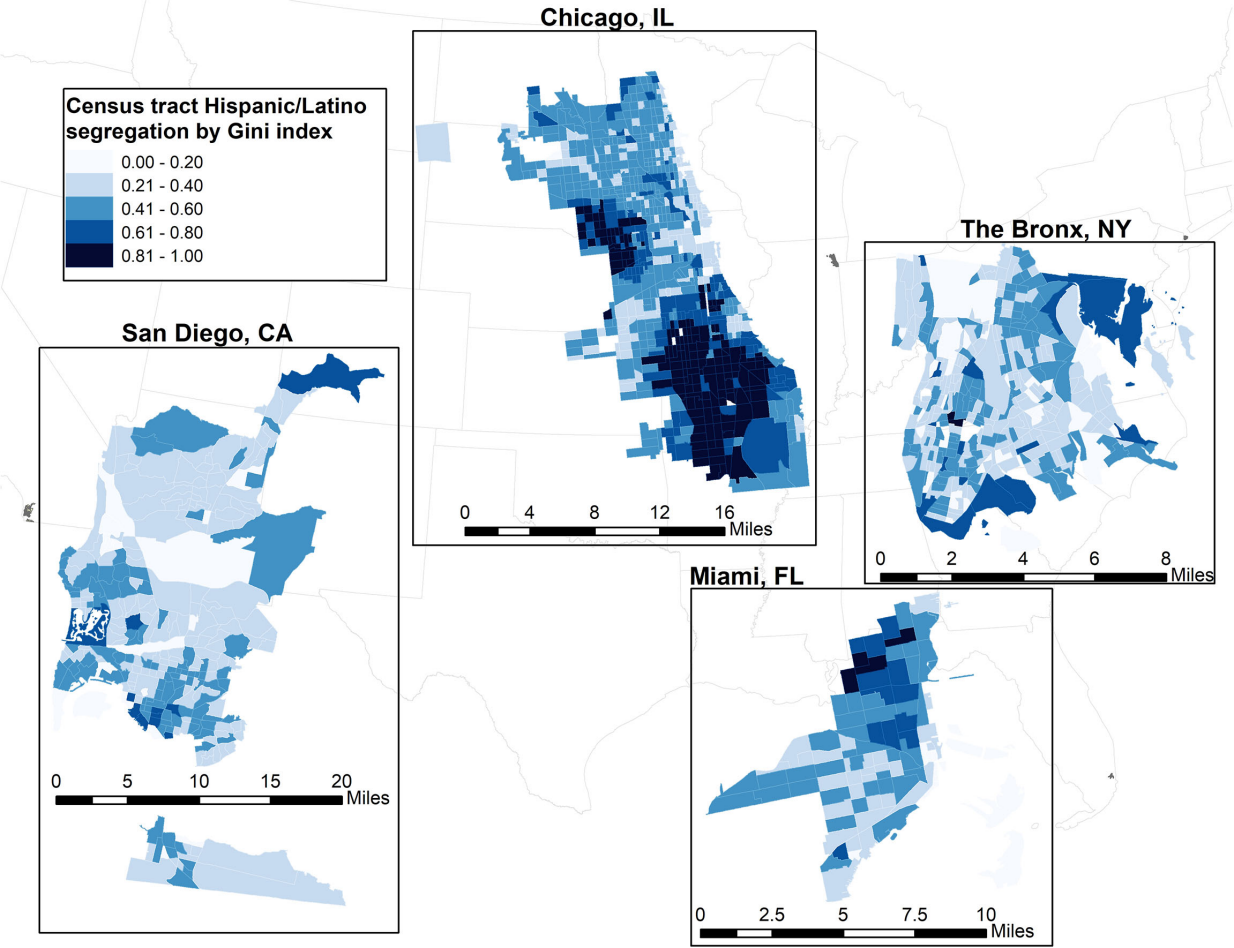
Evenness segregation of Hispanic/Latino census tracts, measured by the Gini index for each study site in the Hispanic Health Community Study/Study of Latinos.

**Figure 2:**
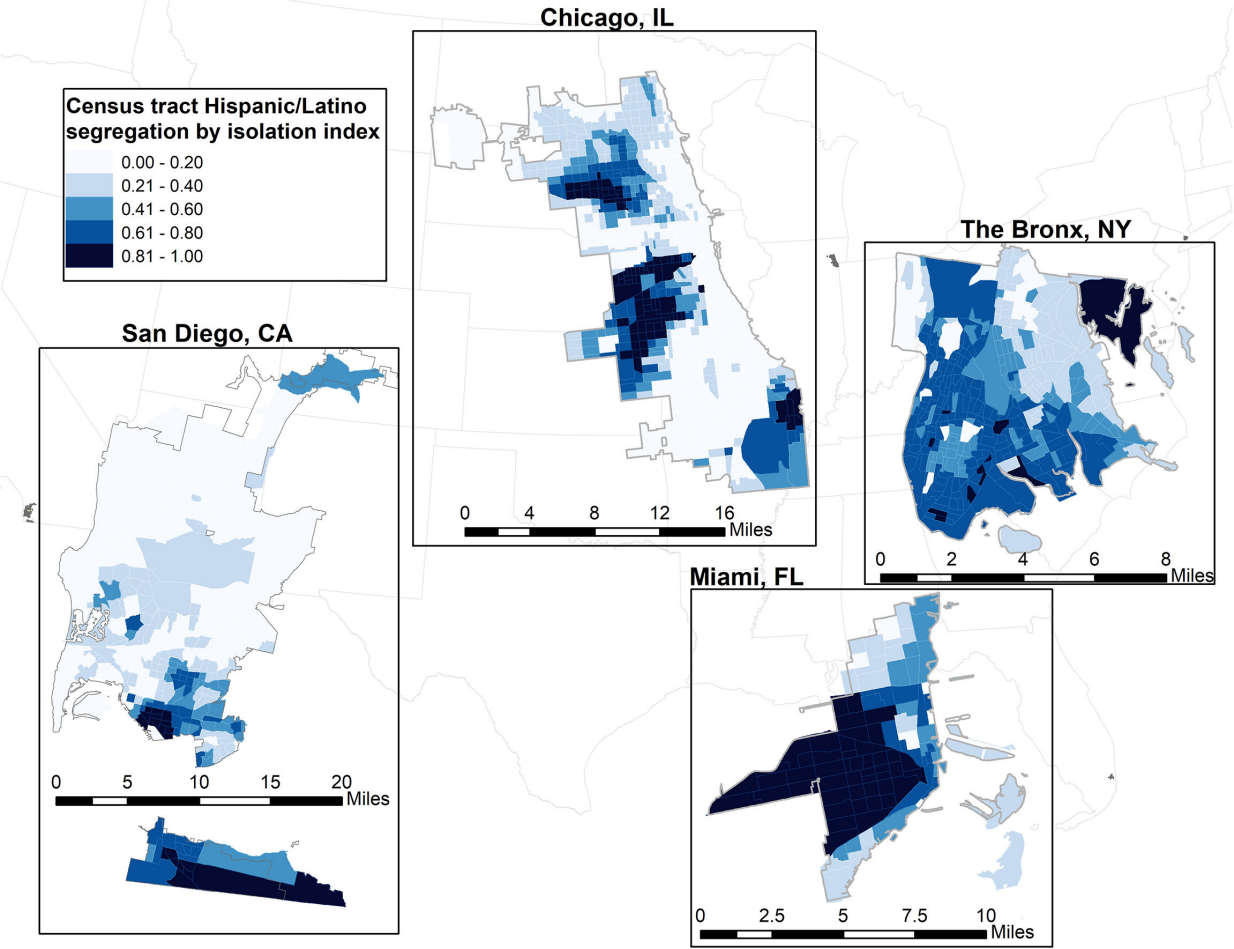
Exposure segregation of Hispanic/Latino census tracts, measured by the Isolation index for each study site in the Hispanic Health Community Study/Study of Latinos.

For our primary analysis, neighborhood segregation was examined using two formal dimensions—evenness and exposure—using 2010 decennial census tract data at the State level (16, 38, 39). The joint effect of evenness and exposure captured hypersegregation. We measured evenness segregation through Gini coefficient of Hispanic/Latino (**Figure 1**) (16, 38). The Gini coefficient measures the variability of Hispanic/Latino residents within the census tract, ranging from 0 to 1 (i.e., with 1 indicating greater segregation). We measured exposure segregation through the isolation index (**Figure 2**) (39). The isolation index ranges from 0 to 1 with higher values suggesting increased probability of interacting with a Hispanic/Latino resident (i.e., greater isolation/segregation). Census-tract level segregation values were calculated based on ethnicity proportions at the block-level according to previous methods (16).

### Neighborhood segregation: Informal/proxy measures

For our secondary analyses, in an effort to compare with prior studies, informal or proxy measures of segregation were examined—Hispanic/Latino density (proportion of adults in a census tract) and racialized economic segregation.

#### Hispanic/Latino density

A widely used proxy for neighborhood segregation is Hispanic/Latino density. For comparability with existing literature and with other formal measures of segregation, we operationalized Hispanic/Latino density using the 2006-2010 American Community Survey data. Higher values indicate higher proportion of Hispanic/Latino residents in the neighborhood.

#### Racialized economic segregation

Using data from the 2006-2010 5-year estimates of the American Community Survey, we calculated the proportional imbalance between affluence and poverty to obtain an Index of Concetration at the Extremes (ICE), which can range from -1 (low racial/ethnic or economic privilege) to 1 (most racial/ethnic or economic privileged). This measure allows us to examine the combined (i.e., racialized economic segregation) and separate influence of concentration of income as well as race/ethnicity. As such, three different types of ICE indices were calculated based on work by Krieger et al., utilizing income data alone, race/ethnicity data alone, and an integration of both income and race/ethnicity data (40, 41). Based on the 20th and 80th percentiles of the national household income distribution of the 2010 Census data, deprived groups were defined as those earning ≥ U.S $25,000 and advantaged groups were those earning ≥ U.S. $100,000.

### ACS guideline adherence score

For comparability with the existing body of literature on adherence, the 2012 ACS Guidelines on Nutrition and Physical Activity for Cancer Prevention, outlined in **Table 1**, were operationalized as a composite score based on previous studies (7, 8).

**Table 1:** The 2012 American cancer society guidelines on nutrition and physical activity for cancer prevention^1^.

1. Achieve and maintain a healthy weight throughout life.
2. Be physically active. Get at least 150 minutes of moderate intensity or 75 minutes of vigorous intensity activity each week (or a combination of these), preferably spread throughout the week.
3. Eat a healthy diet, with an emphasis on plant foods.
3a. Limit how much processed meat and red meat you eat.
3b. Eat at least 21/2 cups of vegetables and fruits each day.
3c. Choose whole grains instead of refined grain products.
3d. If you drink alcohol, limit your intake. Drink no more than 1 drinker day for women or 2 per day for men.

^1^Kushi et al. (3). “American Cancer Society guidelines on nutrition and physical activity for cancer prevention: reducing the risk of cancer with healthy food choices and physical activity.” CA Cancer J Clin 62(1): 30-67.

#### Diet

Diet data came from two 24-hour dietary recalls that assessed intake of specific foods and food groups during the past 12 months (42). The diet components were scored as follows: (1) fruits and vegetables - 1 point for consuming ≥5 servings/day and 0 otherwise; (2) total carotenoids - 0, 1 or 2 points for being in the first, second or third tertile of carotenoid intake; (3) red and processed meat – log transformed, divided into quartiles and assigned scores of 0-3 (lowest quartile = 3); and (4) whole grains, defined as percentage of whole grains consumed (whole grains/total grains x 100) then divided into quartiles and assigned a score of 0-3 (lowest quartile = 0). A final diet score (ranged 0-9) was obtained by summing across the four diet components.

#### Alcohol

Alcohol intake as grams per day, derived from the dietary recall and described in detail previously (43), was considered separately from the diet score. One drink was defined as 14 grams of pure alcohol.

#### Physical activity

Accelerometer-assessed moderate to vigorous physical activity (MVPA) was captured using an Actical accelerometer that participants wore for 7 days to assess frequency, duration, and intensity of their physical activity during that period. Further details of the accelerometry protocol, data cleaning, and derivation are available elsewhere (44).

#### Body mass index

Anthropometric measures (height and weight) were obtained during the baseline visit at each study site. Self-reported weight and height at age 21 years were also collected. Body mass index at enrollment and at age 21 were calculated using the formula kg/m^2^ and categorized as: normal weight (BMI 18.5 to < 25.0 kg/m^2^), overweight (25.0 to < 30.0 kg/m^2^) and with obesity (≥ 30.0-50 kg/m^2^). To capture the ACS guideline of maintenance of a healthy weight throughout life, the BMI scoring incorporated BMI at age 21 when available.

#### Composite adherence score

We categorized the diet score, alcohol intake, physical activity, and BMI into three levels. Behaviors most consistent with criteria received a score of “2” (7-9 diet points; nondrinker; ≥150 mins/week moderate activity or ≥75 mins/week of vigorous; and BMI < 25.0 kg/m^2^ at enrollment *and* at age 21. Behaviors with mid-level concordance received a score of “1” (3-6 diet points; > 0 - ≤1 drink/day for women or 0- ≤2 drink/day for men; >0 to <150 mins/week of moderate activity or >0 to <75 mins/week of vigorous activity; and BMI of 25.0 to <30.0 at enrollment or at age 21). Behaviors with least consistency to guidelines received a score of “0” (0-2 diet points; >1 drink/day for women or >2 drink/day for men; 0 mins/week for physical activity; and BMI ≥ 30 kg/m^2^ at study entry or at age 21). For participants with missing BMI at age 21, only BMI at study entry was used.

Components were summed with possible range of “0” (does not meet recommendations) to “8” (meets all recommendations), and further categorized based on *a priori* cut points used in other studies that included Hispanic/Latino adults (7, 8) as low (0-3), moderate (4-5), and high (6-8) adherence.

### Covariates

We identified potential *a priori* individual and neighborhood level confounders including age categories (18-44, 45-65, >65), education (<high school, high school, some college, ≥college), sex (male, female), employment status (employed, unemployed), marital status (married, otherwise), household income (<$30,000, ≥$30,000, missing), acculturation level (language preference (Spanish, English), birthplace and duration of residence in the U.S. mainland (US born, foreign/US territory born <10 years, foreign/US territory born ≥10 years, missing), Hispanic/Latino heritage, and study site (Miami, San Diego, the Bronx, Chicago). Missing data was coded as the highest level in each categorical covariate.

Neighborhood level confounders included the neighborhood immigrant composition (percent of foreign-born residents in the tract) and the neighborhood deprivation index. The neighborhood deprivation index was calculated according to the approach originally described by Messer et al. (45). Using principal component analysis, we extracted a single factor that represented the shared variance from the following variables: percent of residents with less than a high school diploma, percent of residents with household incomes below 100% of the federal poverty level, percent of residents who are unemployed, and median household income. The index was standardized; increasing values indicated higher neighborhood deprivation.

### Statistical analysis

#### Weights and missing data

We conducted complex survey analysis that accounted for sample weights and a two-stage sampling design from the HCHS/SOL study (46). Models included inverse probability weighting (IPW), due to missing accelerometry data as described previously (47, 48). Briefly, 92.3% (n=15,153) of participants had partial accelerometer data and 77% (N=12,750) had complete data (i.e., = 3 adherent days with > 10 hours of wear time (44). The product of the IPW weight and HCHS/SOL sampling weights were used in models of guideline adherence using accelerometry-measured physical activity which allowed for inferences to the target Hispanic/Latino population. Complex survey designed was accounted for using overall sampling weights; inverse probability weights accounted for missing accelerometer data. We excluded participants with missing data on variables of interest (not mutually exclusive): home addresses (n=316); residing outside of counties of interest (n = 70); accelerometry data (n = 3,933); body mass index at study entry (n = 428); and intake of meat (n = 1,086), grains (n = 1,086), fruits (n = 1,086), vegetables (n = 1,086), nuts and legumes (n= 223), and carotenoids (n = 434). The final analytic sample was 11,957 adults.

#### Model building

Using design-based weighted analyses, we described participant characteristics by ACS guideline adherence. We examined correlations between segregation exposures. We fit weighted multinominal logistic regressions of ACS guideline adherence to estimate relative risk ratios (RRR) and 95% confidence intervals (CI) for a 1-unit increase in neighborhood segregation. Sequential multivariable analyses to control for potential confounders were performed. Model 1 adjusted for individual-level (e.g., age, sex, education, household income, self-identified heritage, study site) covariates. To evaluate the role of segregation, beyond neighborhood deprivation, model 2 additionally included the neighborhood deprivation index. Hypersegregation was examined using joint effects model that examined additive and multiplicative interactions between the dimensions of segregation and guideline adherence outcomes. The proportional odds/parallel lines assumptions were examined in each independent model and because of contradictory results between model fit indices (i.e., Akaike information criterion (AIC) and Bayesian information criterion (BIC)), results from ordinal models (Odds ratio, OR and 95% CI) are also shown. Sensitivity analysis examined associations among never smokers and among participants with complete data for BMI at age 21 at study entry (data not shown). All analysis was conducted in STATA and two-sided tests were considered statistically significant at p<0.05.

## Results

### Descriptive statistics

Differences in sociodemographic characteristics were found by ACS guideline adherence levels (**Table 2**). Overall, 28% of Hispanic/Latino adults were classified as highly adherent to the 2012 ACS guidelines. The majority of Hispanic/Latino adults were ages 18-44 (60%), female (52%), had less than a high school education (32%), had a Spanish-language preference (75%), were US/territory born ≥ 10 years (49%), had health insurance (50%), and had never smoked cigarettes. Overall, the mean BMI at study entry and age 21 were 29.4 and 24.0 kg/m^2^, respectively. Participants engaged in about 150 and 24 minutes/week of moderate and vigorous activity respectively, consumed 4.9 servings/day of fruits and vegetables, 2.1 servings/day of red and processed meats, 22% of whole grains out of total grains consumed per day, and 1.9 servings/week of alcohol.

**Table 2:** Characteristics of U.S. Hispanic/Latinos by ACS guidelines on nutrition and physical activity for cancer prevention categories.

ACS Guideline Adherence Categories^1^		Total	Low Adherence	Moderate Adherence	High Adherence	P^3^
	No. of Participants	11, 957	1,710, 13.7%	7,156, 58.8%	3, 091. 27.5%	
***Demographics***		**Weighted column %**				
Age, %						<0.001
18-44	4,553	60.2%	51.4%	57.4%	70.6%	
45-65	6,384	31.5%	38.2%	33.0%	25.0%	
>65	1,020	8.3%	10.7%	9.6%	4.4%	
Sex						<0.001
Male	4,765	47.8%	41.0%	46.0%	55.2%	
Female	7,192	52.2%	59.0%	54.0%	44.8%	
Education						0.0305
< High school	4,621	32.2%	33.0%	33.0%	30.0%	
High School	3,014	28.3%	27.5%	27.1%	31.0%	
Some college	1,476	12.1%	13.7%	12.5%	10.4%	
≥ College	2,824	27.4%	25.7%	27.2%	28.5%	
Missing	22	0.1%	0.2%	0.1%	0.0%	
Employment status						
Unemployed	5,644	48.0%	54.8%	48.9%	42.8%	<0.001
Employed	6,181	50.6%	44.2%	50.0%	55.2%	
Missing	132	1.4%	1.0%	1.2%	2.1%	
Marital status						
Single, divorced, widowed	8,961	65.7%	69.2%	68.4%	58.0%	<0.001
Married or partnered	2,996	34.3%	30.8%	31.6%	42.0%	
***Acculturation***						
Language preference						0.3251
English	2,179	24.8%	26.0%	23.9%	26.3%	
Spanish	9,778	75.2%	74.0%	76.1%	73.7%	
Place of birth						<0.001
Foreign born in US <10 years	2,759	28.1%	25.9%	27.1%	31.6%	
Foreign born in US >=10 years	7,253	49.2%	53.2%	51.7%	41.8%	
US Born	1,897	22.2%	20.3%	20.7%	26.3%	
Missing	48	0.5%	0.6%	0.5%	0.3%	
***Health-related characteristics***						
Health Insurance						0.0017
Not insured	5,896	50.2%	44.0%	50.7%	52.3%	
Insured	6,061	49.8%	56.0%	49.3%	47.7%	
Smoking status						<0.001
Current	2,116	20.3%	25.3%	19.7%	19.2%	
Former	2,462	17.4%	19.6%	18.9%	13.2%	
Never	7,362	61.9%	55.0%	60.8%	67.6%	
Missing	17	0.4%	0.0%	0.7%	0.1%	
Cancer history						0.0014
No	11,467	96.5%	94.4%	96.5%	97.5%	
Yes	490	3.5%	5.6%	3.5%	2.5%	
***Lifestyle behaviors, mean ± standard error***						
Body size, kg/m2						
Body mass index at age 21 years, n = 9, 614	11,957	24.0 ± 0.1	24.7 ± 0.4	24.2 ± 0.1	23.1 ± 0.1	<0.001
Body mass index at study entry	9,614	29.4 ± 0.11	33.8 ± 0.3	30.3 ± 0.2	25.3 ± 0.1	<0.001
Physical Activity						
Self-reported Leisure time (min/week)						
Moderate	11,897	89.8 ± 4.1	70.0 ± 6.7	85.5 ± 5.7	108.8 ± 7.2	0.001
Vigorous	11,1902	81.4 ± 4.0	46.8 ± 10.4	69.4 ± 4.4	124.2 ± 7.3	<0.001
Self-reported Total (min/week)						
Moderate	11,915	670.1 ± 16.6	482.5 ± 27.6	647.1 ± 20.7	812.5 ± 31.7	<0.001
Vigorous	11,913	291.0 ± 11.0	239.7 ± 38.0	259.1 ± 11.6	384.9 ± 20.6	<0.001
Accelerometry-measured (min/week)						
Moderate	11,957	149.8 ± 2.9	70.1 ± 3.7	132.4 ± 3.1	226.4 ± 5.0	<0.001
Vigorous	11,957	24.1 ± 1.4	4.3 ± 0.6	19.4 ± 1.7	44.0 ± 2.7	<0.001
Diet						
Total energy, kcal/d	11,957	2,065.3 ± 14.7	1,979.7 ± 35.6	1,995.5 ± 18.2	2,257.2 ± 27.2	<0.001
Fruit and vegetables, servings/d	11,957	4.9 ± 0.1	4.0 ± 0.1	4.8 ± 0.1	5.6 ± 0.1	<0.001
Total carotenoids, mg/d	11,957	33.7 ± 0.4	27.2 ± 0.8	32.2 ± 0.4	40.0 ± 0.8	<0.001
Red and processed meat, servings/d	11,957	2.1 ± 0.1	2.11 ± 0.1	2.0 ± 0.1	2.2 ± 0.1	0.487
Whole grains, servings/d	11,957	1.6 ± 0.1	0.5 ± 0.0	1.5 ± 0.1	2.4 ± 0.1	<0.001
Proportion of grains consumed as whole grains	11,957	21.9 ± 0.6	7.5 ± 0.6	21.4 ± 0.7	30.1 ± 1.0	<0.001
Alcohol intake among drinkers (servings/day)	11,957	0.3 ± 0.02	1.1 ± 0.1	0.2 ± 0.02	0.03 ± 0.01	<0.001
Alcohol intake among drinkers (servings/week)	11,957	1.9 ± 0.1	8.0 ± 0.7	1.3 ± 0.1	0.2 ± 0.04	<0.001
***Neighborhood segregation measure^2^***						
Formal measures of segregation						
Evenness dimension	11,957	0.39 ± .004	0.40 ± .006	0.39 ± 0.05	0.39 ± 0.005	0.138
Exposure dimension	11,957	0.76 ± 0.007	0.79 ± 0.01	0.76 ± 0.007	0.75 ± 0.008	<0.001
Proxy measure of segregation						
Economic Segregation	11,957	-0.28 ± 0.01	-0.32 ± 0.02	-0.29 ± 0.01	-0.25 ± 0.02	<0.001
Racial Segregation	11,957	-0.64 ± 0.01	-0.69 ± 0.01	-0.64 ± 0.01	-0.61 ± 0.01	<0.001
Racialized Economic Segregation	11,957	-0.26 ± 0.01	-0.31 ± 0.01	-0.27 ± 0.01	-0.24 ± 0.01	<0.001
Hispanic/Latino Density	11,957	0.74 ± 0.01	0.77 ± 0.01	0.74 ± 0.01	0.72 ± 0.01	<0.001

ACS, American Cancer Society.

^1^The ACS guideline adherence score ranged from 0-8. Data from baseline assessments were used in this analysis.

^2^The evenness dimension of segregation was measured with the Gini coefficient (1 indicates higher segregation); the exposure dimension was measured with the Isolation index (higher values indicate higher segregation); economic segregation measured via Index of Concentration at the Extremes (ICE) for income; racial segregation measured via ICE for race; Racialized economic segregation measured via ICE for income and race (-1 indicates low privilege, +1 indicates higher privilege).

^3^P values derived from Designed-based F tests.

Correlations between neighborhood segregation measures are shown in **Table 3**. Overall, Hispanic/Latino adults lived in low segregated environments based on the evenness dimension (0.39, **Figure 1**) and highly isolated neighborhoods based on the exposure dimension (0.76, **Figure 2**). Hispanic/Latinos tended to reside in neighborhood environments with lower economic and racial privilege (economic segregation = -0.28, racial segregation = -0.64, racialized economic segregation = -0.26).

**Table 3:** Correlations between neighborhood segregation measures in analytical sample.

	(1)	(2)	(3)	(4)	(5)	(6)
(1) Evenness dimension of segregation	1					
(2) Exposure dimension of segregation	0.274	1				
(3) Economic segregation	-0.080	-0.394	1			
(4) Racial segregation	-0.107	-0.853	0.574	1		
(5) Racialized economic segregation	-0.051	-0.585	0.897	0.763	1	
(6) Hispanic/Latino (HL) Density	0.168	0.942	-0.435	-0.933	-0.650	1
(7) Neighborhood deprivation index	0.111	0.194	-0.828	-0.390	-0.671	0.219

On average, adults more adherent to ACS guidelines were younger (age 18-44 at 71%), male (55%), had lower education (31% high school or 30% less than high school), were employed (55%), single (58%), enrolled at the San Diego site (30%), less acculturated (preferred Spanish (74%), foreign born in US <10 years (32%), or in US/territory born >=10 years (42%), of Mexican heritage (46%), not insured (52%), and were never smokers (68%).

Adherence to the ACS guideline and its components varied by Hispanic/Latino heritage (**Table 4**) and study site (**Table 5**). Adults of Mexican heritage and adults enrolled in San Diego had the highest proportion of overall adherence to the ACS guidelines as well as high adherence to the alcohol recommendations. Adults of Mexican heritage and those enrolled in Chicago had the highest proportions of high adherence to the dietary recommendations. Adults of South American heritage and enrolled in Miami had the highest proportion of high adherence to the BMI recommendations. Adults of Puerto Rican heritage and enrolled in the Bronx had the highest proportion of adherence to the physical activity recommendations.

**Table 4:** Proportion of adults meeting the ACS nutrition and physical activity cancer prevention guidelines, by self-reported Hispanic/Latino Heritage, N = 11, 957.

		All Heritage	Dominican	Central American	Cuban	Mexican	Puerto Rican	South American	>1 One Heritage	Missing Heritage	P^8^
	**No. of Participants**	**11,957**	**1,103**	**1,230**	**1,588**	**4,902**	**1,958**	**822**	**333**	**214**	
***Adherence to Guideline Components***											
Alcohol^1,6^											<0.001
Low	528	4.6%	4.0%	3.7%	4.1%	4.9%	4.8%	4.4%	7.1%	16.8%	
Moderate	1,240	11.8%	10.8%	10.9%	21.5%	7.4%	12.6%	9.2%	11.7%	0.0%	
High	10,179	83.6%	85.2%	85.4%	74.4%	87.7%	82.7%	86.3%	81.2%	83.3%	
Dietary^2,6^											
Low	3,856	34.5%	51.7%	35.5%	41.7%	20.2%	46.6%	37.6%	39.9%	65.6%	<0.001
Moderate	7,359	59.9%	47.3%	59.3%	55.4%	70.0%	49.7%	60.0%	57.7%	33.4%	
High	742	83.6%	1.0%	5.2%	2.8%	9.8%	3.7%	2.4%	2.4%	1.0%	
Body Mass Index^3,6^											0.002
Low	3,857	29.7%	33.4%	27.6%	31.2%	27.4%	33.2%	22.6%	35.5%	11.4%	
Moderate	5,609	48.0%	45.7%	48.9%	45.6%	51.0%	45.5%	49.9%	41.5%	45.0%	
High	2,329	22.3%	20.9%	23.5%	23.2%	21.6%	21.3%	27.5%	23.0%	43.7%	
Physical Activity^4,7^											
Low	231	1.73%	1.2%	1.3%	2.9%	1.2%	2.4%	1.8%	0.4%	0.0%	
Moderate	7,548	60.0%	47.8%	58.8%	77.3%	59.6%	50.3%	55.2%	54.7%	38.0%	<0.001
High	4,178	38.3%	51.0%	39.9%	19.9%	39.2%	47.3%	43.0%	44.8%	62.0%	
Guideline Adherence score^5,7^											<0.001
Low	1,710	13.7%	13.5%	12.1%	21.5%	8.6%	17.4%	8.4%	17.5%	9.7%	
Moderate	7,156	58.8%	62.3%	60.3%	60.3%	57.6%	57.2%	58.9%	58.1%	34.9%	
High	3,091	27.5%	23.2%	27.4%	18.3%	33.9%	25.4%	32.7%	24.5%	55.5%	

ACS, American Cancer Society. Data from baseline assessments were used in this analysis.

^1^The alcohol recommendation was operationalized as 2 points for non-drinkers (high adherence) and 1 point for consuming up to 1 or 2 drinks per day for women and men (moderate adherence), respectively, and 0 points if exceeding the alcohol recommendations (low adherence).

^2^The dietary recommendations were operationalized as a summation score, ranging from 0-9 points, across 4 diet components: (1) servings of red and processed meats per day divided into quartiles (Q) and assigned a score of 0-3 (lowest Q = 3); (2) 1 point for consuming ≥5 fruits and vegetables (including nuts and legumes), (3) 1 or 2 points for being in the second or third tertile of total carotenoids, respectively; (4) percentage of whole grains over total grains consumed divided into quartiles and assigned a score of 0-3 (lowest Q =0). Dietary adherence was then classified as low (0-2 diet points), moderate (3-6 diet points) and high (7-9 diet points) adherence.

^3^The body mass index (BMI) recommendation was operationalized as 2 points for maintaining a BMI <25kg/m^2^ at age 21 and at study entry (high adherence), 1 point for maintaining a BMI between 25-30 kg/m^2^ at either time (moderate adherence), and 0 points for BMI ≥30kg/m^2^ at either point (low adherence).

^4^The physical activity recommendations were operationalized using accelerometer measured MVPA where 2 points were given for engaging in ≥150 minutes/week of moderate or ≥75 minutes/week of vigorous activity per week (high adherence), 1 point for MVPA below recommended levels (moderate adherence), 0 points for 0 MVPA (low adherence).

^5^A summation across representing overall guideline adherence across scores for diet, alcohol, BMI and MVPA was calculated and ranged from 0-8. A priori cut offs for guideline adherence were low (score 0-3), moderate (score 4-5), and high (score 6-8) adherence.

Models accounted for ^6^overall complex survey weights or ^7^inverse probability weights for missing accelerometry data.

^8^P values derived from Designed-based F tests.

**Table 5:** Proportion of adults meeting the ACS guidelines on nutrition and physical activity for cancer prevention guidelines, by Study Site,N = 11, 957.

		Bronx, NY	Chicago, IL	Miami, FL	San Diego, CA	P^8^
	No. of Participants	2,966	3,283	2,752	2,956	
***Adherence to Guideline Components***						
Alcohol^1,6^						<0.001
Low	528	3.7%	6.1%	4.0%	5.4%	
Moderate	1,240	9.6%	10.8%	19.3%	6.7%	
High	10,179	86.7%	83.2%	76.7%	87.9%	
Dietary^2,6^						<0.001
Low	3,856	51.1%	20.6%	39.0%	20.2%	
Moderate	7,359	46.4%	70.0%	58.0%	70.6%	
High	742	2.5%	9.4%	3.0%	9.3%	
Body Mass Index^3,6^						<0.001
Low	3,857	33.2%	28.6%	29.6%	26.5%	
Moderate	5,609	46.7%	49.2%	46.0%	50.8%	
High	2,329	20.1%	22.2%	24.3%	22.7%	
Physical Activity^4,7^						<0.001
Low	231	1.4%	1.6%	2.4%	1.4%	
Moderate	7,548	44.1%	60.4%	73.2%	62.7%	
High	4,178	54.6%	38.0%	24.3%	35.9%	
Guideline Adherence Score^5,7^						<0.001
Low	1,710	14.2%	11.3%	18.6%	8.9%	
Moderate	7,156	59.4%	56.5%	60.0%	58.2%	
High	3,091	26.4%	32.2%	21.5%	33.0%	

ACS, American Cancer Society. Data from baseline assessments were used in this analysis.

^1^The alcohol recommendation was operationalized as 2 points for non-drinkers (high adherence) and 1 point for consuming up to 1 or 2 drinks per day for women and men (moderate adherence), respectively, and 0 points if exceeding the alcohol recommendations (low adherence).

^2^The dietary recommendations were operationalized as a summation score, ranging from 0-9 points, across 4 diet components: (1) servings of red and processed meats per day divided into quartiles (Q) and assigned a score of 0-3 (lowest Q = 3); (2) 1 point for consuming ≥5 fruits and vegetables (including nuts and legumes), (3) 1 or 2 points for being in the second or third tertile of total carotenoids, respectively; (4) percentage of whole grains over total grains consumed divided into quartiles and assigned a score of 0-3 (lowest Q = 0). Dietary adherence was then classified as low (0-2 diet points), moderate (3-6 diet points) and high (7-9 diet points) adherence.

^3^The body mass index (BMI) recommendation was operationalized as 2 points for maintaining a BMI <25kg/m^2^ at age 21 and at study entry (high adherence), 1 point for maintaining a BMI between 25-30 kg/m^2^ at either time (moderate adherence), and 0 points for BMI ≥30kg/m^2^ at either point (low adherence).

^4^The physical activity recommendations were operationalized using accelerometer measured MVPA where 2 points were given for engaging in ≥150 minutes/week of moderate or ≥75 minutes/week of vigorous activity per week (high adherence), 1 point for MVPA below recommended levels (moderate adherence), 0 points for 0 MVPA (low adherence).

^5^A summation across representing overall guideline adherence across scores for diet, alcohol, BMI and MVPA was calculated and ranged from 0-8. A priori cut offs for guideline adherence were low (score 0-3), moderate (score 4-5), and high (score 6-8) adherence.

Models accounted for ^6^overall complex survey weights or ^7^inverse probability weights for accelerometer data.

^8^P values derived from Designed-based F tests.

### Associations for dimensions of segregation and guideline adherence

In fully adjusted multinominal regression, we found no association between the evenness dimension or exposure dimension of segregation and ACS guideline adherence category (**Table 6**). Furthermore, no associations were found after including both evenness and exposure dimensions in the joint effects models.

**Table 6:** Multinomial logistic regression models for the association between neighborhood segregation measures and adherence to the ACS guidelines^1^ on nutrition and physical activity for cancer prevention.

	Model 1^3,5^Relative Risk Ratio (95% CI)				Model 2^4,5^Relative Risk Ratio (95% CI)			
	Low	Moderate	High	P	Low	Moderate	High	P^6^
**Formal measures of segregation^2^**								
*Main effects*								
Evenness dimension	1.00	0.94 (0.86, 1.03)	0.93 (0.85, 1.03)		1.00	0.94 (0.86, 1.04)	0.96 (0.86, 1.06)	
Exposure dimension	1.00	0.95 (0.88, 1.04)	0.95 (0.86, 1.03)		1.00	0.95 (0.88, 1.04)	0.98 (0.89, 1.09)	
*Joint Effects*								
Evenness, while controlling for Exposure	1.00	0.95 (0.87, 1.05)	0.94 (0.85, 1.05)		1.00	0.95 (0.86, 1.05)	0.96 (0.86, 1.07)	
Exposure, while controlling for Evenness	1.00	0.96 (0.88, 1.04)	0.96 (0.87, 1.05)		1.00	0.96 (0.88, 1.04)	0.99 (0.89, 1.09)	
Evenness x Exposure, while controlling for main effects	1.00	1.02 (0.99, 1.05)	1.02 (0.98, 1.05)	0.552	1.00	1.02 (0.99, 1.05)	1.02 (0.98, 1.05)	0.583
Evenness x Exposure, without main effects	1.00	1.00 (0.99, 1.01)	1.00 (0.99, 1.00)	0.764	1.00	1.00 (0.99, 1.01)	1.00 (0.99, 1.00)	0.862
**Proxy measures of segregation^2^**								
Economic Segregation	1.00	1.08 (0.69, 1.70)	1.47 (0.86, 2.49)		1.00	1.00 (0.60, 1.66)	1.24 (0.71, 2.19)	
Racial Segregation	1.00	1.31 (0.84, 2.05)	1.49 (0.92, 2.41)		1.00	1.30 (0.82, 2.06)	1.23 (0.72, 2.08)	
Racialized Economic Segregation	1.00	1.35 (0.81, 2.24)	1.94 (1.01, 3.70)		1.00	NA	NA	
Hispanic/Latino Density	1.00	0.60 (0.28, 1.26)	0.54 (0.25, 1.17)		1.00	0.60 (0.28, 1.29)	0.72 (0.31, 1.68)	

ACS, American Cancer Society; CI, Confidence Interval, NA, Not Applicable.

^1^The ACS guideline adherence score ranged from 0-8. A priori cut offs for guideline adherence were low (score 0-3), moderate (score 4-5), and high (score 6-8). Data from baseline assessments were used in this analysis.

^2^The evenness dimension of segregation was measured with the Gini coefficient; the exposure dimension was measured with the Isolation index; economic segregation measured via Index of Concentration at the Extremes (ICE) for income; racial segregation measured via ICE for race; Racialized economic segregation measured via ICE for income and race.

^3^Model 1 was adjusted for individual level covariates: age (<45, 45-65, >65), sex (male, female), education (<HS, HS, Some College, College, Missing), income (less than $30,000, $30,000 or more, missing), marital status (married, otherwise), insurance status (yes, no), place of with combined with years in the US (US born, Foreign born and <10 years in US, Foreign born and 10+ years in US, Missing), Language preference (Spanish, English), Hispanic/Latino heritage (Mexican, Dominican, Puerto Rican, Cuban, Central American, South American, Other or More than 1 heritage, Missing), study site (the Bronx, Chicago, Miami, San Diego).

^4^Model 2 also adjusted for neighborhood level covariates as follows: models for evenness, racial segregation, and HL density included neighborhood deprivation index, while models for evenness, exposure, and economic segregation adjusted for neighborhood immigrant concentration).

^5^All models accounted for complex survey design using inverse probability weights.

^6^P values for multiplicative models were calculated using loglikelihood ratio tests comparing nested models with and without interaction effects.

### Associations for dimensions of segregation and individual components of the guidelines

In fully adjusted multinominal regression models, there was evidence of an association between exposure segregation (i.e., higher residential isolation) and lower likelihood of having moderate vs. low adherence to the alcohol recommendations (**Table 6**). Evenness segregation associated with lower likelihood of having high vs. low adherence to the physical activity guidelines. In a series of multinominal regression models examining joint effects (additive and multiplicative, **Table 7**), we found evidence that residence in hypersegregated neighborhoods associated with moderate vs. low adherence to the alcohol, dietary and BMI recommendations and with both moderate and high vs. low adherence to the physical activity recommendations.

**Table 7:** Multinomial logistic regression models for the association between neighborhood segregation measures and adherence to the individual components of the ACS guidelines^1^ on nutrition and physical activity for cancer prevention.

	Alcohol^3,4^				Diet^3,4^			Body Mass Index^3,4^			Physical Activity^3,5^		
	Low	Moderate	High	P^6^	Moderate	High	P^6^	Moderate	High	P^6^	Moderate	High	P^6^
		Odds Ratio and (95% CI)											
**Formal measures of segregation^2^**													
*Main effects*													
Evenness dimension	1.00	1.05 (0.89, 1.24)	0.99 (0.85, 1.15)		1.02 (0.95, 1.09)	1.00 (0.88, 1.14)		1.01 (0.94, 1.08)	1.05 (0.96, 1.15)		0.80 (0.63, 1.01)	0.73 (0.57, 0.94)	
Exposure dimension	1.00	0.86 (0.75, 0.98)	0.90 (0.78, 1.04)		1.07 (1.01, 1.14)	1.08 (0.97, 1.20)		1.00 (0.94, 1.07)	1.03 (0.95, 1.12)		0.97 (0.82, 1.15)	0.92 (0.77, 1.10)	
*Joint Effects*													
Evenness, while controlling for Exposure	1.00	1.07 (0.91, 1.26)	1.01 (0.87, 1.17)		1.01 (0.94, 1.09)	0.99 (0.86, 1.13)		1.01 (0.95, 1.08)	1.05 (0.96, 1.15)		0.80 (0.64, 1.00)	0.74 (0.58, 0.94)	
Exposure, while controlling for Evenness	1.00	0.85 (0.74, 0.98)	0.90 (0.78, 1.05)		1.07 (1.01, 1.14)	1.08 (0.97, 1.21)		1.00 (0.94, 1.07)	1.03 (0.95, 1.11)		0.98 (0.84, 1.15)	0.94 (0.80, 1.12)	
Evenness x Exposure, while controlling for main effects	1.00	1.01 (0.95, 1.07)	1.00 (0.95, 1.05)	0.776	1.01 (0.98, 1.03)	0.99 (0.94, 1.04)	0.672	1.03 (1.01, 1.06)	1.02 (0.99, 1.05)	0.030	1.03 (0.95, 1.12)	1.02 (0.94, 1.11)	0.415
Evenness x Exposure, without main effects	1.00	1.00 (0.98, 1.01)	1.00 (0.99, 1.01)	0.148	1.00 (1.00, 1.01)	0.99 (0.98, 1.00)	0.712	1.00 (1.00, 1.00)	1.00 (1.00, 1.01)	0.234	.99 (0.98, 1.01)	0.99 (0.97, 1.00)	0.727
**Proxy measures of segregation**													
Economic Segregation	1.00	1.78 (0.89, 3.59)	0.99 (0.46, 2.13)		1.22 (0.85, 1.76)	1.13 (0.61, 2.10)		1.53 (1.07, 2.19)	1.72 (1.08, 2.73)		0.47 (0.11, 1.97)	0.43 (0.09, 2.01)	
Racial Segregation	1.00	1.89 (0.90, 3.96)	1.44 (0.69, 2.99)		0.76 (0.56, 1.04)	1.09 (0.56, 1.80)		1.15 (0.84, 1.58)	1.02 (0.68, 1.51)		1.22 (0.47, 3.16)	1.41 (0.51, 3.87)	
Racialized Economic Segregation	1.00	2.34 (0.89, 6.11)	1.32 (0.48, 3.62)		1.10 (0.72, 1.68)	1.29 (0.53, 3.17)		1.75 (1.14, 2.71)	2.08 (1.18, 3.67)		0.67 (0.20, 2.30)	0.59 (0.15, 2.36)	
Hispanic/Latino Density	1.00	0.40 (0.13, 1.25)	0.66 (0.22, 2.03)		1.57 (0.97, 2.55)	1.25 (0.52, 3.01)		0.81 (0.49, 1.34)	1.06 (0.57, 1.98)		0.68 (0.19, 2.40)	0.51 (0.13, 1.99)	

ACS, American Cancer Society; CI, Confidence Interval.

^1^The ACS guideline adherence score ranged from 0-8. A priori cut offs for guideline adherence were low (score 0-3), moderate (score 4-5), and high (score 6-8). Data from baseline assessments were used in this analysis.

^2^The evenness dimension of segregation was measured with the Gini coefficient; the exposure dimension was measured with the Isolation index; economic segregation measured via Index of Concentration at the Extremes (ICE) for income; racial segregation measured via ICE for race; Racialized economic segregation measured via ICE for income and race.

^3^All models were adjusted for individual level covariates: age (<45, 45-65, >65), sex (male, female), education (<HS, HS, Some College, College, Missing), income (less than $30,000, $30,000 or more, missing), marital status (married, otherwise), insurance status (yes, no), place of with combined with years in the US (US born, Foreign born and <10 years in US, Foreign born and 10+ years in US, Missing), Language preference (Spanish, English), Hispanic/Latino heritage (Mexican, Dominican, Puerto Rican, Cuban, Central American, South American, Other or More than 1 heritage, Missing), study site (the Bronx, Chicago, Miami, San Diego); and neighborhood level covariates as follows: models for evenness, racial segregation, and HL density included neighborhood deprivation index, while models for evenness, exposure, and economic segregation adjusted for neighborhood immigrant concentration).

^4^Models accounted for complex survey design using overall sampling or ^5^inverse probability weights for missing accelerometer data.

^6^P values for multiplicative models were calculated using loglikelihood ratio tests comparing nested models with and without interaction effects.

### Secondary analyses: Associations for proxies of neighborhood segregation and guideline adherence

Racialized economic segregation was associated with higher likelihood of having high vs. low overall guideline adherence (**Table 6**). Residence in areas with either higher economic segregation or higher racialized economic segregation was associated positively with the likelihood of having moderate or high vs. low adherence to the BMI recommendations. Other proxies of segregation (racial segregation or Hispanic/Latino density) did not associate with overall guideline adherence or its components.

Results from ordered logistic regression models are shown in **Table 8**.

**Table 8:** Ordered logistic regression models for the association between neighborhood segregation measures and adherence to the individual components of the ACS^1^ guidelines on nutrition and physical activity for cancer prevention, N = 11, 957.

	Model 1^3^, Odds Ratio (95% CI)	P^5^	Model 2^4^ Odds Ratio (95% CI)	P^5^
**Formal measures of segregation** ^2^				
*Main effects*				
Evenness dimension	0.97 (0.91, 1.02)		0.98 (0.93, 1.04)	
Exposure dimension	0.97 (0.93, 1.03)		1.00 (0.94, 1.06)	
*Joint Effects*				
Evenness, while controlling for Exposure	0.97 (0.92, 1.03)		0.98 (0.92, 1.05)	
Exposure, while controlling for Evenness	0.98 (0.93, 1.03)		1.00 (0.95, 1.06)	
Evenness x Exposure, while controlling for main effects	1.01 (0.99, 1.03)	0.441	1.01 (0.99, 1.03)	0.469
Evenness x Exposure, without main effects	1.00 (0.99, 1.00)	0.489	1.00 (0.99, 1.00)	0.622
**Proxy measures of segregation^2^**				
Economic Segregation	1.31 (0.96, 1.78)		1.19 (0.87, 1.63)	
Racial Segregation	1.23 (0.93, 1.63)		1.08 (0.80, 1.46)	
Racialized Economic Segregation	1.50 (1.00, 2.26)			
Hispanic/Latino Density	0.73 (0.48, 1.13)		0.89 (0.56,1.43)	

ACS, American Cancer Society; CI, Confidence Interval.

^1^The ACS guideline adherence score ranged from 0-8. A priori cut offs for guideline adherence were low (score 0-3), moderate (score 4-5), and high (score 6-8). Data from baseline assessments were used in this analysis.

^2^The evenness dimension of segregation was measured with the Gini coefficient; the exposure dimension was measured with the Isolation index; economic segregation measured via Index of Concentration at the Extremes (ICE) for income; racial segregation measured via ICE for race; Racialized economic segregation measured via ICE for income and race.

^3^Model 1 was adjusted for individual level covariates: age (<45, 45-65, >65), sex (male, female), education (<HS, HS, Some College, College, Missing), income (less than $30,000, $30,000 or more, missing), marital status (married, otherwise), insurance status (yes, no), place of with combined with years in the US (US born, Foreign born and <10 years in US, Foreign born and 10+ years in US, Missing), Language preference (Spanish, English), Hispanic/Latino heritage (Mexican, Dominican, Puerto Rican, Cuban, Central American, South American, Other or More than 1 heritage, Missing), study site (the Bronx, Chicago, Miami, San Diego).

^4^Model 2 also adjusted for neighborhood level covariates as follows: models for evenness, racial segregation, and HL density included neighborhood deprivation index, while models for evenness, exposure, and economic segregation adjusted for neighborhood immigrant concentration). All models accounted for complex survey design using inverse probability weights.

^5^P values for multiplicative models were calculated using loglikelihood ratio tests comparing nested models with and without interaction effects.

## Discussion

In this large and diverse population of U.S. Hispanic/Latino adults, we examined whether formal and proxy measures of neighborhood segregation were associated with adherence to the 2012 ACS Guidelines on Nutrition and Physical Activity for Cancer Prevention. In our analysis, formal (e.g., evenness and exposure) measures of segregation were suggestive of a 2-7% lower odds of guideline adherence for every 1 unit increase in segregation. Segregation was also associated with several ACS guideline components. In multiplicative models, there was evidence of an association between hypersegration and BMI. Based on our proxy measures, individuals living in more affluent areas (economic segregation) were 28%-47% more likely to meet the BMI recommendations, whereas Hispanic/Latino adults residing in areas with both greater racial and economic privilege (i.e. more residents identifying with the White race and affluence) were almost 2 times more likely to meet them.

Our study expands a growing body of evidence that attempts to understand the role of neighborhood segregation on energy balance and cancer related inequities. Extant cancer research has focused on the role of neighborhood deprivation on cancer preventive behaviors (49) and cancer risk and outcomes (50), fewer studies have examined segregation, and among these, most relied on proxy measures of segregation (18).

### Exposure dimension and ACS guideline adherence

The literature on segregation and cancer-related outcomes (examined with formal measures) is mixed (51, 52), focusses on multiple sequential and interacting segregation mechanisms as well as possible moderating effects of segregation not captured in our work. For example, our cross-sectional analysis is suggestive of possible mediating effects of neighborhood poverty given the large observed change in direction and attenuated magnitude of some estimates after we adjusted for the neighborhood deprivation index, consistent with the body of literature showing that neighborhood segregation leads to concentrated poverty (53–56). Our study also adds to a large but mixed body of literature on the role of neighborhood segregation or ethnic enclave on dietary patterns (33, 57, 58). Our findings are consistent with a body of literature showing that segregated poor communities are more likely to have increased exposure to alcohol and tobacco outlets and advertisements. Segregation, regardless of neighborhood racial/ethnic composition, has been associated with higher number of alcohol (59, 60) outlets.

The exposure dimension of segregation measures the probability of interaction with other members of the same racial/ethnic group. In our study, Hispanic/Latino adults resided in highly segregated neighborhoods (isolation index of 0.78, with >0.6 indicative of high segregation). High exposure to members of racial/ethnic groups that exhibit poor lifestyle behaviors and outcomes (i.e., limited exposure to healthier groups) may lead to poor lifestyle behaviors and health at the individual level (61). For example, Hispanic/Latinos are at high risk of sedentary behaviors (62, 63), and barriers include discouragement from peers and cultural norms (27). We found that only evenness segregation, after adjusting for isolation, was associated with a lower likelihood of meeting the physical activity guidelines. Although, it is important to note that other individual (e.g., fatigue, limited time), environmental (e.g., safety, lack of resources), and financial (e.g., cost) level factors related to segregation are strong barriers to physical activity among Hispanic/Latino adults (27, 64, 65).

### Evenness and adherence to ACS guidelines

When racial/ethnic groups are unevenly distributed, thereby becoming isolated into smaller pockets across a given geographic space, access to health promoting resources become concentrated in neighboring concentrated White communities and health inequities arise. Studies suggest that evenness segregation may not be associated with adverse health unless it is accompanied by isolation (i.e., hypersegregation) (66–68). Our findings counter this, in that evenness segregation alone was negatively associated with lower odds of meeting physical activity guidelines and when considered simultaneously, evenness segregation remained negatively associated with higher levels of physical activity.

### Racialized economic concentration at the extremes

Our findings on racialized economic segregation are, in part, consistent with literature showing that Hispanic/Latino adults residing in segregated communities were more likely to be economically disadvantaged compared to those residing in non-segregated communities (54, 69, 70). In turn, they experienced decreased access to resources that enabled adoption and maintenance of cancer preventive behaviors (physical activity, walkable, open spaces, affordable quality foods) (61, 71).

We found that racialized economic concentration was not associated with overall guideline adherence but was associated with meeting the BMI recommendations. While we are unaware of any other study linking ICE indices to health behaviors, our findings align with prior studies demonstrating a link between racialized economic segregation and adverse health outcomes (41, 72). In these studies, economic and race-based segregation was associated with higher BMI among Hispanic/Latino adults of Mexican heritage (72) and worse cancer outcomes (50, 73, 74). Similarly, we found that Hispanic/Latinos residing in neighborhoods with greater racialized economic segregation (i.e., higher economic and/or racial privilege) were more likely to meet the recommendations for BMI and alcohol intake, but less likely to meet them for diet. Our findings suggest that both race/ethnicity and socioeconomic standing have a significant role in place-based stratification (75). Among Hispanic/Latino adults, socioeconomic gains or increased assimilation do not always translate to spatial assimilation; as residential gains for Hispanic/Latino of diverse heritage (i.e., adults reporting mixed-race and ethnicity, Black Hispanic adults) are achieved at a higher cost compared to their White counterparts (70, 76, 77). Additionally, among Hispanic/Latinos adults, the poverty rates of non-White neighbors are a major driver of poverty concentration, which explains the importance of capturing the interaction of class- and race- based segregation (78) at the neighborhood level.

## Strengths and limitations

Our study has notable strengths and some limitations. We used data from a large and diverse sample of US. Hispanic/Latino adults (46), generalizable to Hispanic/Latino adults in Chicago, IL; San Diego, CA; Miami, FL and the Bronx, NY. We conceptualized segregation using multiple formal measures as well as novel proxies that integrate both dimensions of structural racism (segregation and poverty). We used objective measures of physical activity, and dietary data were derived from questionnaires designed and validated in our study population to capture traditional and culturally specific foods. Lastly, we adjusted for a range of important confounders (e.g., acculturation, heritage) for Hispanic/Latino populations that are known to contribute to variations in lifestyle behaviors.

Limitations of our study include the cross-sectional nature of the data that limits causal inferences due to temporality of the measures, the possibility of unmeasured confounders such as skin color (20) and lack of residential history (75, 79). Future studies could examine time varying associations, account for changes in participant’s residential mobility, and explore the role of segregation at other known important neighborhood levels (e.g. county or block) (66).

This analysis evaluated the adherence to ACS guidelines using data collected between 2008-2011 and prior to the 2012 publication of the ACS guidelines. While our study does not evaluate guideline adherence over time as the guidelines became more widely recognized and implemented, our findings suggest that adoption and long-term maintenance of the guidelines has likely faced significant challenges in segregated neighborhood environments. Consideration of social and structural environments will be critical to the successful adoption of cancer preventive behaviors among Hispanic/Latino adults who reside in segregated neighborhoods or ethnic enclaves. Future studies should examine whether guideline adherence among Hispanic/Latinos has changed over time in light of the revised ACS recommendations published in June 2020.

## Conclusion

Hispanic/Latino adults live in neighborhoods with high concentrations of racial/ethnic and economic segregation (33). Therefore, the lack of resources to engage in healthful behaviors in these neighborhoods translates to fewer opportunities to adopt and maintain healthful lifestyles and meet the guidelines on nutrition and physical activity for cancer prevention. Public health policies and interventions that specifically focus on segregated neighborhoods has the potential to improve the adoption and maintenance of cancer preventive behaviors among Hispanic/Latino adults.

## Data availability statement

Data are maintained by the individual Hispanic Health Community Study/Study of Latinos study centers and collaborative studies coordinating center at the University of North Carolina Chapel Hill and are available upon submitting a proposal to be approved by the HCHS/SOL publications committee. Requests to access the datasets should be directed to https://sites.cscc.unc.edu/hchs/New%20Investigator%20Opportunities.

## Ethics statement

The studies involving human participants were reviewed and approved by The Hispanic Community Health Study/Study of Latinos, San Diego State University. The patients/participants provided their written informed consent to participate in this study.

## Author contributions

Conceptualization, MSP, SFC, YM, and JP; Methodology, MSP, CMP, LCG, and JP; Coding, MSP, CMP, and JP; Formal Analysis, MSP and CMP; Resources, GAT, JP; Writing – Original Draft Preparation, MSP, CMP, and JP; Writing – Review and Editing, MSP, CMP, SFC, YM, GT, LCG, KRE, MLD, LH, DS-A, BJ, LA-S, and JP; Supervision, GAT, JP; Funding Acquisition, JP. All authors contributed to the article and approved the submitted version.
